# Prevention of V.D.

**Published:** 1921-06-25

**Authors:** Ettie A. Rout

**Affiliations:** (late Hon. Sec. N.Z. Volunteer Sisters). London


### THE EDITOR'S LETTER-BOX.

**Correspondence on all subjects is invited, but we cannot in any way be responsible for the opinions expressed by our correspondents, who must give their name and address as a guarantee of good faith, but not necessarily for publication. Correspondents are reminded that brevity of style and conciseness of statement greatly facilitate early insertion.**

### Prevention of V.D.

*To the Editor of* The Hospital.

Sir,—Will you please permit me to endorse the arguments so well stated in your thoughtful article entitled "The Public Well-being," published in this week's Hospital? They agree almost entirely with the following opinion, which was expressed in 1915 by the A.D.M.S. of the Australian Forces in Egypt (Colonel Sir James Barrett) and afterwards published by Colonel Barrett and Lieutenant Deane in their book on the work of the Australian Army Medical Corps in Egypt (1914-1915), pp. 127-128:

"The essence of the problem was learnt by a Brigadier-General who visited a number of young educated men in one of the camps, and asked them for their viewpoint on the subject. Their answer was that which every medical officer knows full well: that many men were influenced by the appeals which had been made to them, but that a percentage have indulged in this way throughout their adult life, and intended to continue to do so irrespective of anything medical officers, chaplains, or generals may say to them. It is this fundamental position which every reformer must face. So long as a sufficient number of men determine to adopt this policy, and so long as there is a sufficient number of women prepared to cater for them, the problem of venereal disease will continue to be acute in every country.

"The opinion has been expressed elsewhere that the world will not be rendered more or less moral by the abolition of venereal disease, and instruction in the mode of preventing infection should be an essential part of education. Because people are immoral, there is no reason why they should acquire gonorrhœa or syphilis. If the *lex talionis* is to be enforced, the logical way to deal with the matter is to refuse treatment to all the infected, and to let them die or become disabled. But the most thoroughgoing Puritan shrinks from adopting so terrible a policy. One method or the other, however, must be adopted—there can be no half-way house."

And Colonel Sir James Barrett set forth his reasons for strongly advocating disinfection methods. He gave evidence before the Inter-Departmental Committee, and the whole of his evidence and that of Sir William Osler and others, in favour of disinfection, was omitted from the Report. Officially, England has adopted the half-way-house compromise of treating disease rather than preventing infection—a policy which is equivalent to muzzling all the dogs after they have gone mad.

Yours faithfully,

Ettie A. Rout

(late Hon. Sec. N.Z. Volunteer Sisters).

London, June 15, 1921.

